# The Effects of Three Chlorhexidine-Based Mouthwashes on Human Osteoblast-Like SaOS-2 Cells. An In Vitro Study

**DOI:** 10.3390/ijms22189986

**Published:** 2021-09-15

**Authors:** Giulia Brunello, Kathrin Becker, Luisa Scotti, Dieter Drescher, Jürgen Becker, Gordon John

**Affiliations:** 1 Department of Oral Surgery, University Clinic of Düsseldorf, 40225 Düsseldorf, Germany; giulia.brunello@med.uni-duesseldorf.de (G.B.); luisa.c.scotti@gmail.com (L.S.); juergen.becker@med.uni-duesseldorf.de (J.B.); gordon.john@med.uni-duesseldorf.de (G.J.); 2 Department of Neurosciences, University of Padua, 35128 Padua, Italy; 3 Department of Orthodontics, University Clinic of Düsseldorf, 40225 Düsseldorf, Germany; dieter.drescher@med.uni-duesseldorf.de; 4 Dental Practice, 46147 Oberhausen, Germany

**Keywords:** antiseptic, bone, cetylpyridinium chloride, chlorhexidine, mouthrinse, peri-implantitis, periodontitis

## Abstract

Several decontamination methods for removing biofilm from implant surfaces during surgical peri-implantitis treatment have been reported, including the intraoperative usage of chlorhexidine (CHX)-based antiseptics. There is a lack of information on possible adverse effects on bone healing. The study aimed to examine the impact of three CHX-based mouthwashes on osteoblast-like cells (SaOS-2) in vitro. Cells were cultured for three days in 96-well binding plates. Each well was randomly treated for either 30, 60 or 120 s with 0.05% CHX combined with 0.05% cetylpyridinium chloride (CPC), 0.1% CHX, 0.2% CHX or sterile saline (NaCl) as control. Cell viability, cytotoxicity and apoptosis were assessed at day 0, 3 and 6. Cell viability resulted in being higher in the control group at all time points. At day 0, the CHX 0.2 group showed significantly higher cytotoxicity values compared to CHX 0.1 (30 s), CHX + CPC (30 s, 60 s and 120 s) and control (60 s and 120 s), while no significant differences were identified between CHX + CPC and both CHX 0.1 and NaCl groups. All test mouthwashes were found to induce apoptosis to a lower extent compared to control. Results indicate that 0.2% CHX presented the highest cytotoxic effect. Therefore, its intraoperative use should be carefully considered.

## 1. Introduction

Peri-implantitis is a multifactorial bacteria-induced pathology affecting the peri-implant tissues, leading to a progressive reduction of the supporting bone and, subsequently, to implant loss if left untreated [1,2].

While a non-surgical mechanical debridement might be resolutive in the case of peri-implant mucositis, it seems to have limited efficacy for the management of peri-implantitis [3,4]. Although the non-surgical approach represents a fundamental step in the initial treatment of peri-implantitis, in cases of recurrence of bleeding and suppuration, it has to be followed by surgical therapy, which allows a better access for an effective removal of the biofilm from the contaminated implant surfaces [5]. To this aim, several mechanical and chemical techniques have been proposed; however, no particular decontamination protocol has been demonstrated to be superior [5,6,7].

Mouthwashes can be used as adjunctive measures to the mechanical elimination of bacteria through surgical debridement [8]. Among these, chlorhexidine (CHX) is one of the most commonly used products due to its high antibacterial properties [5,9]. However, its beneficial effect is controversial. In a randomized controlled clinical trial on the surgical treatment of advanced peri-implantitis, a 0.2% solution of chlorhexidine digluconate did not exhibit any beneficial effect over the mechanical implant surface decontamination alone at both 1- and 3-year follow-up [10,11]. This is consistent with previous findings in animal models [12]. Furthermore, socket rinsing with CHX has also been proposed, but its effect is still controversial, as some authors reported impairment of wound healing while others reported a reduced rate of alveolitis [13,14].

Several concerns have been raised regarding the potential tissue toxicity of these agents. Numerous studies have investigated the cytotoxicity of CHX on different cells, including fibroblasts, osteoblasts, myoblasts and epithelial cells [15,16,17,18,19,20,21,22,23,24]. In particular, for these clinical applications, CHX-based solutions would be in direct contact with the bone and the connective tissues without the protective barrier of the intact epithelium, thus increasing the risk of cytotoxicity [16,21]. Regarding osteoblasts, 0.1% CHX has been reported to rapidly induce morphological changes and cell damage in human osteoblasts already after an incubation time of one minute [25]. In John et al. [20], 0.2% CHX was found to be cytotoxic for SaOS-2 cells. In a study investigating the effect of CHX on the same cell line, cell viability was reduced in dose- and time-dependent manners [26]. A dose-dependent CHX cytotoxicity was also observed in other in vitro studies [15,17,23].

Furthermore, CHX-induced perioperative hypersensitivity has been extensively reported in the literature [27]. Although severe reactions are rarely observed in relation to mouthwashes, rinsing with an open flap might increase the risk of their occurrence.

To reduce the CHX-related side effects, shorter exposure time and/or lower concentration of CHX alone or in combination with additional compounds have been recommended. The combination CHX and cetylpyridinium chloride (CPC) has been demonstrated to be effective when used as an adjunct to oral hygiene for patients in supportive periodontal care [28,29,30], as well as in cases of peri-implant mucositis [31,32]. A 0.12% CHX + 0.05% CPC solution was found to reduce bacterial load to a greater extent than mechanical debridement alone in respective peri-implantitis treatment [33] and exhibited similar clinical, radiographic and microbiological outcomes as compared to an alcohol containing 0.2% CHX [34]. Nevertheless, whether its additional use translates into enhanced clinical outcomes remains to be clarified.

In a recent study by our team [35], contrary to saline, two commercially available CHX-based mouthwashes (i.e., 0.05% CHX + 0.05% CPC and 0.1% CHX) were found to be effective in the reduction of living bacteria in oral biofilms attached to micro-rough titanium surfaces. Following a 60 s exposure to the mouthwashes, no significant difference was found between the two groups in bacteria viability after 24 as well as 48 h of in situ plaque collection.

Taking into consideration the remarkable antibacterial properties exhibited by a 0.05% CHX + 0.05% CPC mouthwash and the well-documented cytotoxicity associated with antiseptics containing CHX at higher concertation, the relevant clinical question arose of whether it can be safely used as adjunctive in the surgical treatment of peri-implantitis.

Although numerous studies have investigated the effect of different concentrations of CHX on osteoblasts, to the best of our knowledge, the effects of a low-concentration CHX solution containing CPC on osteoblasts had not been explored yet. Therefore, the aim of this study was to evaluate in vitro the effects of three commercially available mouthwashes containing CHX at different dilutions, alone or in combination with CPC, on osteoblast-like cells by examining cell viability, cytotoxicity and apoptosis.

## 2. Results

Results on cell viability, cytotoxicity and apoptosis are reported below. No cell culture was lost due to microbial contamination.

### 2.1. Cell Viability

**Figure 1:**
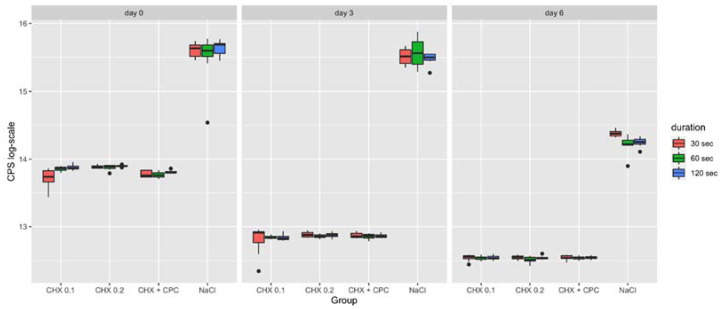
Boxplot representing the cell viability of SaOS-2 cells following the different treatment procedures (i.e., CHX 0.1, CHX 0.2, CHX + CPC and NaCl for 30, 60 and 120 s) at day 0, 3 and 6. Data are expressed in counts per second (CPS).

The highest cell viability values were predictably detected in the control group (NaCl) at all time points, as shown in Figure 1. Unexpectedly and not in accordance with the descriptive analysis illustrated in the boxplot, no significant difference was identified between CHX 0.2 and NaCl groups at day 0 (30 s, 60 s and 120 s) as well as at day 3 (120 s) (Table 1). The *p*-values presented in Table 1 are Bonferroni-corrected. The uncorrected *p*-values were <0.05 in all these cases but one (30 s day 0: *p* = 0.083).

Within all test groups, application time did not affect cell viability, except for CHX 0.1 at day 0. Indeed, significant differences were observed between 30s and the longer application times (i.e., 30 s vs. 60 s; 30 s vs. 120 s).

Only at day 0, statistically significant differences were found between the test groups. In detail, CHX 0.2 presented higher values compared to CHX 0.1 and CHX + CPC after an application time of 30 s and 120 s, respectively.

### 2.2. Cytotoxicity

At day 0, the highest cytotoxicity was detected in the CHX 0.2 group, which presented significantly higher values compared to CHX 0.1 (30 s), CHX + CPC (30 s, 60 s and 120 s) and control (60 s and 120 s). Lower cytotoxicity was exhibited by the CHX 0.1 as compared to NaCl after an application time of 30 s, while the opposite was observed after 120 s (*p* < 0.05 and *p* < 0.01, respectively). For all the exposure times, no significant differences were found between CHX + CPC and CHX 0.1, as well as between CHX + CPC and the control. Moreover, at day 0, the application time was not found to be determinant within the CHX 0.2 group. An increased cytotoxicity dependent on the application time was observed in both CHX 0.1 (60 s > 30 s and 120 s > 30 s) and CHX + CPC (120 s > 30 s). By contrast, within the NaCl, results are inverted, with longer application times associated to a lower cytotoxicity compared to 30 s exposure.

**Figure 2:**
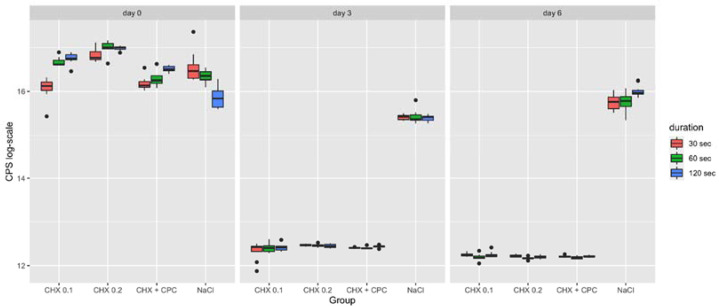
Boxplot representing the cytotoxicity on SaOS-2 cells following the different treatment procedures (i.e., CHX 0.1, CHX 0.2, CHX + CPC and NaCl for 30, 60 and 120 s) at day 0, 3 and 6. Data are expressed in counts per second (CPS).

As evidenced in the graph (Figure 2), at day 3 and day 6, a similar situation was observed, with the highest values recorded in the control group compared to the others. Contrary to day 0, application time was found not to be relevant in the majority of cases. Likewise, for cell viability test, in contrast with what was reported in the boxplot, no significant difference was identified between CHX 0.2 and NaCl at day 3 (30 s and 60 s) and between CHX 0.1 and NaCl at day 6 (30 s) (Table 2). The *p*-values presented in Table 2 are Bonferroni-corrected. The respected uncorrected *p*-values were <0.05 in all three cases.

### 2.3. Apoptosis

**Figure 3:**
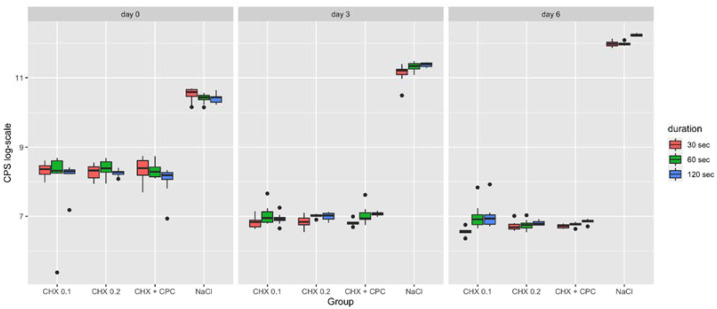
Boxplot representing the apoptosis of SaOS-2 cells following the different treatment procedures (i.e., CHX 0.1, CHX 0.2, CHX + CPC and NaCl for 30, 60 and 120 s) at day 0, 3 and 6. Data are expressed in counts per second (CPS).

At all time points, the highest apoptotic levels were registered in the NaCl control group (Figure 3). As reported in Table 3, within each treatment and control group, at day 0 the application time was not found to significantly influence the apoptotic effect on SaOS-2 cells. At day 3, significant differences in apoptosis were observed between the 30 s and 120 s application times both in the NaCl and CHX + CPC. On the other hand, at day 6, significant differences were registered within all groups but one (i.e., CHX 0.2).

Unexpectedly and not in accordance with what was reported in the boxplot (Figure 3), there was no statistically significant difference between NaCl and CHX + CPC groups at day 3 (120 s) and at day 6 (30 s). The *p*-values presented in Table 3 are Bonferroni-corrected. The uncorrected *p*-values were <0.05 in both cases.

## 3. Discussion

The purpose of this study was to determine the impact of three commercially available mouthwashes containing chlorhexidine (CHX) at different concentrations, alone or in combination with cetylpyridinium chloride (CPC), on osteoblast-like cells (SaOS-2) cultured for 2 h, 3 days and 6 days after different exposure times to the mouthwashes tested in this study (i.e., 30 s, 60 s and 120 s).

Among the tested mouthwashes, the highest cell viability values were predictably recorded in the NaCl (control) group at all three time points, and for all application times. Except for day 0, in which CHX 0.2 showed higher values than CHX 0.1 at 30 s application time as well as higher values than CHX + CPC at an application time of 120 s, cell viability was comparable among the tested mouthwashes. The application time did not reveal any effect on cell viability within the test groups except for CHX 0.1 at day 0.

Besides cell viability, the triplex assay utilized in the present study allowed for exploring the SaOS-2 death mechanism induced by the different treatment procedures. Contrary to apoptosis, which is characterized by cell membrane integrity, its disruption and the subsequent release of the cytoplasmic contents into the surrounding tissue occur in case of necrosis [36,37].

Hence, in virtue of the different morphological features of these two cellular death mechanisms, it was possible to assess the cytotoxicity by means of a fluorogenic proteolytic biomarker that is released from cells that have lost their membrane integrity. The exposure to CHX 0.2 resulted in significantly higher cytotoxicity levels at day 0 compared to CHX 0.1 (30 s), CHX + CPC (30 s, 60 s and 120 s) and NaCl (60 s and 120 s). No significant differences were found between CHX + CPC and both CHX 0.1 and NaCl for all the application times. Interestingly, CHX + CPC as well as CHX 0.1 exhibited a time-dependent cytotoxicity on SaOS-2, whereas an inverse correlation between application time and cytotoxic effect was observed in the NaCl group at day 0. This finding can hardly be explained, as longer rinsing procedures were expected to be associated with higher cellular stress. Nevertheless, as emerged from cell viability assay, well recovery of the cells was evidenced in the control group at day 3 and 6. By contrast, the low cytotoxicity levels in all the test groups at these time points might be attributed to the early death of a great amount of SaOS-2 once in contact with CHX-based agents.

Caspase activation is considered a hallmark of programmed death, or apoptosis [38]. In the current study, caspase-3/7 substrates were utilized for the detection of the activity of these two effector caspases. In a previous study of this group [20], utilizing the same assessment method and cell line (i.e., SaOS-2) to test the in vitro properties of antimicrobial agents, a different control was adopted, i.e., pure water instead of NaCl. Regardless of the type of control, in both studies, higher apoptosis values were detected in the presence of the control compared to CHX-based ones. The low apoptotic levels registered among the test groups might be attributed to the dominant cytotoxic effect of the mouthwashes, while the results detected in the control group might be due to common environmental stress, especially after longer culture time, related to cell confluence, increased amount of waste products and reduced nutrition medium [39].

A fundamental condition for the successful treatment of peri-implantitis is the re-osseointegration of the implants. One of the determinant factors influencing this process, i.e., the re-osseointegration, consists in the effective decontamination of dental implants. To this aim, several approaches have been proposed, with no proven long-term clinical advantage of one method over the others [5,40]. Chemical agents can be used intraoperatively, alone or in combination with other methods, to eliminate bacterial biofilm adhering to the exposed implant surfaces. Indeed, peri-procedural rinsing with CHX has also been recommended for implant surgery in order to reduce the bacterial load [41]. Furthermore, rinsing with CHX after periodontal and implant surgery has been correlated with a significant reduction in plaque and bleeding as compared to placebo [42]. Besides their antimicrobial properties, these products should not exert a detrimental effect on the surrounding tissues, and eventual residues should not compromise the cellular response to the decontaminated surfaces [43]. Re-osteointegration largely depends on the initial cell response at the cell-implant interface. The main cells responsible for new bone apposition are osteoblasts and their precursors; therefore, assessing the effect of different mouthwashes on these cells is particularly relevant for the proposed clinical application.

Prior in vitro research has demonstrated the cytotoxicity of CHX on both osteoblastic and osteoblastic-like (e.g., SaOS-2) cell lines. In a previous paper by our group using a similar study design [20], at day 0, CHX 0.2 exhibited the highest cytotoxicity on SaOS-2, especially after 120 s of exposure, with significantly higher values compared to the taurolidine 2% and the pure water group. In Giannelli et al. [26], exposure to CHX induced a decrease in SaOS-2 cell viability in a dose- and time-dependent manner, while in the present study, the differences between the groups (different CHX concentration/application time) were not pronounced. Similar to our investigation, CHX-based mouthwashes were able to induce both apoptosis and necrosis. The treatment with 0.2% CHX also induced a drastic reduction of viability of both SaOS-2 and bone marrow mesenchymal stromal cells seeded onto titanium disks as compared to untreated cells [44]. Interestingly, CHX-induced cell damage resulted in being attenuated by rinsing with PBS, and even more if followed by air drying. In Vörös et al., 0.1% CHX was found to cause cell damage on human osteoblasts already after an incubation time of one minute [25]. The viability of murine osteoblast precursor cells significantly decreased when exposed to 0.12% CHX as compared to the control, irrespectively of the application time ranging from 30 s to 4.5 min [45].

The cytotoxic profile of CHX was also corroborated at lower concentrations. Osteoblast survival rate 48 h after an exposure to CHX (0.002%) was significantly reduced as compared to the control for all the exposure times (i.e., 1 m, 2 m and 3 m) (Liu et al., 2018). The low viability levels registered in this last work could have been ascribed to the relatively short culture time, masking the regenerative capacity of the cells over time. Therefore, in a recent study investigating the effect of different antiseptic solutions, a longer observation time was selected as in our paper [46]. The cytotoxic effect of CHX was confirmed also at a low concentration (0.05%), with human osteoblast cells failing to recover over the course of 5 days.

To the best of our knowledge, no other paper has previously investigated the effect of a 0.05% CHX + 0.05% CPC solution on osteoblast-like cells in vitro. However, the current study presents some limitations. Firstly, it was confined to a laboratory setting and the obtained results may not correspond to the oral environment, as a monolayer cell culture model cannot fully represent the bone tissue exposure to the antiseptic agents. Osteoblast-like cells were here directly exposed to the mouthwashes, while in vivo they reside within the mineralized bone tissue, which may reduce the permeability and the adsorption of the chemicals. Many aspects cannot be investigated in vitro, including the dilution of the mouthwashes in the fluids present in the oral cavity, the immunological response of the organism, as well as the tissue alterations resulting from the pathology itself [25]. In the present work, a two-dimensional (2D) system was chosen due to the high reproducibility of the experimental results and the ease of culture maintenance. Nevertheless, the morphology as well as the functions of cells grown as a monolayer attached to a glass or plastic surface resulted in being altered compared to those in the natural environment [47,48]. Despite the higher costs and technical difficulties, three-dimensional (3D) cell culture models have gained increasing interest owing to their closer resemblance to the in vivo microenvironment [47,49]. Furthermore, bone repair is a complex process which involves the well-orchestrated interactions between different cells and signals [50]. Microvascular circulation is considered a key component during tissue repair, and the lack of angiogenesis or its inhibition has been reported to hamper bone healing [51]. Newly formed vessels not only supply nutrients and oxygen to meet the local metabolic demands, but also produce inflammatory and injury-induced angiocrine signals, which contribute to guiding bone regeneration [52]. Therefore, 3D co-cultures of osteoblasts and endothelial cells or concurrent multi-lineage differentiation of stem cells might be considered for future studies, prior to in vivo preclinical investigations or human clinical trials.

It is worth mentioning that human tissues usually present a higher tolerance for antiseptic agents compared to monolayer tissue cultures [24]. Indeed, higher regenerative potential is observed in vivo, where the recruitment of osteoprogenitors, hematopoietic stem cells and immune cells plays a fundamental role in tissue regeneration and remodeling [50]. Moreover, the fast growing of cells on a plastic support may further contribute to cell damage, as testified by the high cytotoxicity and apoptotic values reported in the control group at day 3 and 6. When resective surgical treatment of peri-implantitis was combined with surface decontamination with a 0.12% CHX + 0.05% CPC solution, a reduction of the anaerobic bacterial load was observed as compared to a placebo solution [33]. The significant reduction in bacterial load did not translate into an overall clinical or radiographical benefit. However, no detrimental effect was associated with the antiseptic agent. As a consequence, a CHX + CPC solution containing an even lower concentration of CHX could represent a safe antiseptic for this specific application.

Finally, implant surface characteristics have been demonstrated to affect cell response. Therefore, it would be interesting to investigate the effect of the mouthwashes on cells seeded onto different implant surfaces. Pre-treatment of the implant surfaces, simulating commonly applied clinical procedures such as implantoplasty, and different rinsing times with PBS or water after mouthwash application might also be determinant.

All of the mouthwashes tested here caused irreversible SaOS-2 cell damage, as confirmed by the low viability values and the respective low cytotoxicity and apoptotic levels registered at day 3 and 6. The main differences among the tested treatment procedures were observed at day 0, when overall the CHX 0.2 solution was found to exert a higher cytotoxic effect as comparted to the other mouthwashes. While a time-related effect on cell recovery and death was not noticed in the majority of the cases in all the experiments, at day 0 shorter application times were associated to lower cell cytotoxicity in both the CHX 0.1 and CHX + CPC group. It can be deduced that both these products could be considered for intraoperative usage, especially for a short rinsing time, while long application time and the exposure to CHX at the standard concentration of 0.2% should be avoided. How the present findings could be translated into a clinical situation remains to be clarified.

## 4. Materials and Methods

### 4.1. Cell Culture

Osteoblast-like cells (SaOS-2 cells) were seeded on sterile 96-well binding cell-culture plates (Costar 9102, Kennebunk, ME, USA). Following the protocol described in John et al. (John et al., 2014), 10,000 SaOS-2 cells (Acc 243, fourth passage, German Collection of Microorganisms and Cell Cultures GmbH, Braunschweig, Germany) were cultured for 3 days in 200 µL of high-glucose Dulbecco’s Modified Eagle Medium (DMEM, Sigma-Aldrich, Merck Group, St. Louis, MO, USA) supplemented with 10% fetal bovine serum (Sigma-Aldrich) and 1% penicillin/streptomycin (Gibco Invitrogen, Darmstadt, Germany) at a temperature of 37 °C, 95% of humidity and 5% CO_2_.

### 4.2. Treatment Procedure

After 3-day cell culture, a total of 288 wells were randomly assigned to the following treatment groups: 0.05% CPC + 0.05% CHX (PERIO-AID^®^ Active Control, Dentaid^®^ GmbH, Barcelona, Spain) (CPC + CHX), 0.1% CHX (Chlorhexamed^®^ Fluid 0.1%, GlaxoSmithKline Consumer Healthcare GmbH & Co. KG, Bühl, Germany), 0.2% CHX (Chlorhexamed^®^ Forte 0.2%, GlaxoSmithKline Consumer Healthcare GmbH & Co. KG), and sterile saline (NaCl) as control. In the attempt to replicate in vitro the situation of a mouthwash, nutrition medium was removed before the treatment and cells were gently rinsed with phosphate buffered saline (PBS, Sigma-Aldrich). Three treatment times (i.e., 30, 60 and 120 s) were tested in each of the four groups.

Test and control mouthwashes were removed, the wells were gently rinsed with PBS and 200 µL of high-glucose DMEM was applied per well. Two hours (day 0), 3 days and 6 days after the treatment procedure with the mouthwashes, cell viability, cytotoxicity and apoptosis were assessed. In the 6-day groups, the nutrition medium was changed at day 3. Before performing the tests, the nutrition medium was removed and the wells were gently rinsed with PBS.

For each application time and assessment time point, 8 wells per product were examined.

### 4.3. Cell Viability, Cytotoxicity and Apoptosis

The effect of different treatment procedures on cell viability, cytotoxicity and apoptosis was determined by means of a triplex assay (ApoTox-Glo™ Triplex Assay, Promega, Mannhein, Germany) following manufacturer’s instructions.

Firstly, cell viability and cytotoxicity were assessed simultaneously by fluorometry, measuring two protease activities. A viability/cytotoxicity reagent, containing both glycyphenylalanyl-aminofluorocoumarin (GF-AFC) and bis-alanylalanyl-phenylalnyl-rhodamine 100 (AAF-R110), was utilized. GF-AFC is a cell-permeant peptide which enters intact living cells where it is converted into amino fluorocoumarin (AFC), generating a fluorescent signal proportional to the amount of living cells. AAF-R110 is a cell-impermeant peptide, which is converted by dead-cell protease in rhodamine 110 (R100), when the protease is released in the culture medium due to the loss of cell membrane integrity. The metabolic products can be detected simultaneously, owing to the different mission spectra (AFC in green and R110 in red). Thereafter, for apoptosis, caspase-3/7 activity was measured by adding a luminogenic caspase-3/7 substrate, which can be evaluated via the production of a luminescent signal proportional to the amount of caspase activity present.

All signal measurements were performed using a luminometer/fluorometer (Victor 2030, PerkinElmer, Rodgau, Germany). Results were expressed in counts per second (CPS).

### 4.4. Statistical Analysis

Statistical evaluation was performed using the software R [53]. For each time point, application time and mouthwash, boxplots were created for descriptive purposes. The Kruskal-Wallis test, post hoc multiple comparison test and Bonferroni method for *p*-value adjustment were used to assess statistical differences in cell viability, cytotoxicity and apoptosis among the three treatment groups per time point, and adjusted *p*-values were reported. The results were considered significant at *p* < 0.05.

## 5. Conclusions

Further studies are needed to determine the impact of the different products and rinsing times on wound healing when they are used intraoperatively, in direct contact with the bone. Besides the safety of the rinsing procedure, their efficacy in terms of bacterial load reduction, improved bone healing and decreased peri-implantitis recurrences should also be investigated. It would also be important to evaluate the clinical effects of peri-incisional rinsing and postoperative dressings containing CHX-based solutions.

Future research could also be tailored to the investigation of different rinsing protocols in similar contexts, such as extraction socket rinsing or other surgical procedures in which a full thickness mucoperiosteal flap is raised, exposing the bone to the oral cavity.

## Figures and Tables

**Table 1:** Cell viability. A multiple Kruskal-Wallis test was performed to compare the groups at each time point (i.e., at day 0, 3 and 6), and in the case of significance, a post hoc multiple comparison test with Bonferroni *p*-value adjustment was performed. The adjusted *p*-values from post hoc test are reported and labeled as follows: * *p* < 0.05, ** *p* < 0.01, *** *p* < 0.001.

Grouping Variable	Comparator 1	Comparator 2	*p*-Value (Day 0)	*p*-Value (Day 3)	*p*-Value (Day 6)
CHX 0.1	30 s	60 s	0.040 *	-	-
	30 s	120 s	0.005 **	-	-
	60 s	120 s	1.000	-	-
CHX 0.2	30 s	60 s	-	-	-
	30 s	120 s	-	-	-
	60 s	120 s	-	-	-
CHX + CPC	30 s	60 s	-	-	-
	30 s	120 s	-	-	-
	60 s	120 s	-	-	-
NaCl	30 s	60 s	-	-	0.003 **
	30 s	120 s	-	-	1.000
	60 s	120 s	-	-	0.008 **
30 s	CHX 0.1	CHX 0.2	0.033 *	1.000	1.000
	CHX 0.1	CHX + CPC	1.000	1.000	1.000
	CHX 0.1	NaCl	0.000 ***	0.006 **	0.007 **
	CHX 0.2	CHX + CPC	0.141	1.000	1.000
	CHX 0.2	NaCl	0.499	0.007 **	0.004 **
	CHX + CPC	NaCl	0.000 ***	0.001 **	0.002 **
60 s	CHX 0.1	CHX 0.2	1.000	1.000	1.000
	CHX 0.1	CHX + CPC	0.310	1.000	1.000
	CHX 0.1	NaCl	0.017 *	0.001 **	0.007 **
	CHX 0.2	CHX + CPC	0.054	1.000	1.000
	CHX 0.2	NaCl	0.123	0.008 **	0.000 ***
	CHX + CPC	NaCl	0.000 ***	0.009 **	0.016 *
120 s	CHX 0.1	CHX 0.2	1.000	0.733	1.000
	CHX 0.1	CHX + CPC	0.274	1.000	1.000
	CHX 0.1	NaCl	0.014 *	0.000 ***	0.003 **
	CHX 0.2	CHX + CPC	0.024 *	1.000	1.000
	CHX 0.2	NaCl	0.185	0.050	0.004 **
	CHX + CPC	NaCl	0.000 ***	0.004 **	0.006 **

**Table 2:** Cytotoxicity. A multiple Kruskal-Wallis test was performed to compare the groups at each time point (i.e., at day 0, 3 and 6), and in the case of significance, a post hoc multiple comparison test with Bonferroni *p*-value adjustment was performed. The adjusted *p*-values from post hoc test are reported and labeled as follows: * *p* < 0.05, ** *p* < 0.01, *** *p* < 0.001.

Grouping Variable	Comparator 1	Comparator 2	*p*-Value (Day 0)	*p*-Value (Day 3)	*p*-Value (Day 6)
CHX 0.1	30 s	60 s	0.011 *	-	-
	30 s	120 s	0.000 ***	-	-
	60 s	120 s	0.967	-	-
CHX 0.2	30 s	60 s	-	-	0.250
	30 s	120 s	-	-	0.014 *
	60 s	120 s	-	-	0.819
CHX + CPC	30 s	60 s	0.916	0.231	-
	30 s	120 s	0.004 **	0.030 *	-
	60 s	120 s	0.085	1.000	-
NaCl	30 s	60 s	0.014 *	-	1.000
	30 s	120 s	0.001 **	-	0.027 *
	60 s	120 s	1.000	-	0.071
30 s	CHX 0.1	CHX 0.2	0.000 ***	0.695	1.000
	CHX 0.1	CHX + CPC	1.000	1.000	0.733
	CHX 0.1	NaCl	0.043 *	0.002 **	0.068
	CHX 0.2	CHX + CPC	0.002 **	0.073	1.000
	CHX 0.2	NaCl	1.000	0.241	0.002 **
	CHX + CPC	NaCl	0.123	0.000 ***	0.000 ***
60 s	CHX 0.1	CHX 0.2	0.559	0.695	1.000
	CHX 0.1	CHX + CPC	0.054	1.000	1.000
	CHX 0.1	NaCl	0.141	0.001 **	0.009 **
	CHX 0.2	CHX + CPC	0.000 ***	0.472	1.000
	CHX 0.2	NaCl	0.000 ***	0.131	0.001 **
	CHX + CPC	NaCl	1.000	0.000 ***	0.006 **
120 s	CHX 0.1	CHX 0.2	0.420	1.000	1.000
	CHX 0.1	CHX + CPC	0.947	1.000	1.000
	CHX 0.1	NaCl	0.006 **	0.001 **	0.033 *
	CHX 0.2	CHX + CPC	0.008 **	1.000	1.000
	CHX 0.2	NaCl	0.000 ***	0.017 *	0.001 **
	CHX + CPC	NaCl	0.373	0.005 **	0.002 **

**Table 3:** Apoptosis. A multiple Kruskal-Wallis test was performed to compare the groups at each time point (i.e., at day 0, 3 and 6), and in the case of significance, a post hoc multiple comparison test with Bonferroni *p*-value adjustment was performed. The adjusted *p*-values from post hoc test are reported and labeled as follows: * *p* < 0.05, ** *p* < 0.01, *** *p* < 0.001.

Grouping Variable	Comparator 1	Comparator 2	*p*-Value (Day 0)	*p*-Value (Day 3)	*p*-Value (Day 6)
CHX 0.1	30 s	60 s	-	-	0.005 **
	30 s	120 s	-	-	0.003 **
	60 s	120 s	-	-	1.000
CHX 0.2	30 s	60 s	-	-	-
	30 s	120 s	-	-	-
	60 s	120 s	-	-	-
CHX + CPC	30 s	60 s	-	0.078	0.915
	30 s	120 s	-	0.002 **	0.003 **
	60 s	120 s	-	0.730	0.074
NaCl	30 s	60 s	-	0.121	1.000
	30 s	120 s	-	0.014 *	0.004 **
	60 s	120 s	-	1.000	0.009 **
30 s	CHX 0.1	CHX 0.2	1.000	1.000	0.320
	CHX 0.1	CHX + CPC	1.000	1.000	0.173
	CHX 0.1	NaCl	0.004 **	0.004 **	0.000 ***
	CHX 0.2	CHX + CPC	1.000	1.000	1.000
	CHX 0.2	NaCl	0.001 **	0.006 **	0.026 *
	CHX + CPC	NaCl	0.010 *	0.002 **	0.056
60 s	CHX 0.1	CHX 0.2	1.000	1.000	1.000
	CHX 0.1	CHX + CPC	1.000	1.000	1.000
	CHX 0.1	NaCl	0.006 **	0.002 **	0.048 *
	CHX 0.2	CHX + CPC	1.000	1.000	1.000
	CHX 0.2	NaCl	0.006 **	0.011 *	0.001 **
	CHX + CPC	NaCl	0.002 **	0.002 **	0.002 **
120 s	CHX 0.1	CHX 0.2	1.000	1.000	0.878
	CHX 0.1	CHX + CPC	1.000	0.472	1.000
	CHX 0.1	NaCl	0.020 *	0.000 ***	0.036 *
	CHX 0.2	CHX + CPC	1.000	1.000	1.000
	CHX 0.2	NaCl	0.006 **	0.004 **	0.000 ***
	CHX + CPC	NaCl	0.000 ***	0.068	0.006 **

## Data Availability

Data will be provided upon reasonable request.
